# Advances in Clinical Practices: A Cross-Disciplinary Review of Nursing, Pharmacy, and Medical Science Contributions

**DOI:** 10.7759/cureus.86408

**Published:** 2025-06-20

**Authors:** Karthi R., J. Jabaseeli Gladies Mary, Arul Valan P., T. Darthi Tamilmozhi, R. Arthi, A. S. Malathi

**Affiliations:** 1 Department of Nursing, E.S. College of Nursing, The Tamil Nadu Dr. M. G. Ramachandran (MGR) Medical University, Villupuram, IND; 2 Department of Nursing, Dr. Kumaraswmi Health Centre College of Nursing, The Tamil Nadu Dr. M. G. Ramachandran (MGR) Medical University, Villupuram, IND

**Keywords:** advanced clinical practice, clinical pharmacy innovation, interprofessional collaboration in healthcare, interprofessional education, precision medicine integration

## Abstract

Global healthcare requires faster integration between nursing practice and medical science, together with pharmacy expertise, which allows distinct but compatible skills of all domains to work together. This assessment provides a comprehensive analysis of how interprofessional collaboration among nursing staff and medical scientists, and pharmacists creates positive impacts on healthcare innovation and patient success, and current healthcare delivery challenges. The article explores Advanced Practice Registered Nurse (APRN) service expansion through chronic disease leadership and patient-centered care practices, and pharmacist integration of pharmacogenomics with drug delivery systems and precision medicine (PM), and minimally invasive regenerative treatments. The combination of pharmaceutical clinicians who work with multidisciplinary healthcare teams alongside nurse safety quality implementation leads to reduced medication errors and enhanced patient care processes. The foundation of maintaining collaborative healthcare systems depends on interprofessional education (IPE) for healthcare providers and continuous training. The evaluation performed by this review depends on recent data and expert panels to create complete thematic findings, but its narrative synthesis approach brings specific analysis challenges. Successful clinical practice of the future requires health professionals to work together with shared goals and genuine professional respect to provide equivalent services to patients.

## Introduction and background

Healthcare continues to change constantly through medical developments, technological breakthroughs, as well as a better understanding of health and diseases. The development of healthcare depends heavily on the contributions made by nursing professionals, along with those from the pharmacy and medical science fields. Major healthcare breakthroughs tend to develop when individual fields collaborate through joint efforts that unite their particular areas of knowledge [1]. This review evaluates recent developments in these fields by discussing their separate advantages and demonstrates how collaborative care enhances patient results [2].

Nursing practice has extended its boundaries through the evolving roles of Advanced Practice Registered Nurses (APRNs), such as nurse practitioners and clinical nurse specialists who now deliver medical services typically provided by physicians [3]. Advanced roles in nursing practice improve care access, specifically for people who need medical attention most while experiencing chronic health concerns. Nurses successfully manage hypertension and hyperlipidemia in diabetic patients while performing follow-up care for rheumatoid arthritis patients [4]. The profession of nursing combines research outcomes with evidence-based practices (EBPs) for symptom control and care development, which reinforces the person-centered focus of nursing care [5].

The pharmacy now serves as a clinical healthcare profession that prioritizes patient needs. Pharmacists give healthcare teams essential information about pharmacokinetics, together with drug interaction management and personalized therapy strategies [6]. The healthcare teams now depend on pharmacists for medication reconciliation and disease management, and antimicrobial stewardship (AMS) activities [7]. Advances in pharmacogenomics (PGx) and biologics continue to reshape treatment strategies, while pharmacists contribute to improved outcomes through education, adherence support, and cost-effective care models.

**Figure 1:**
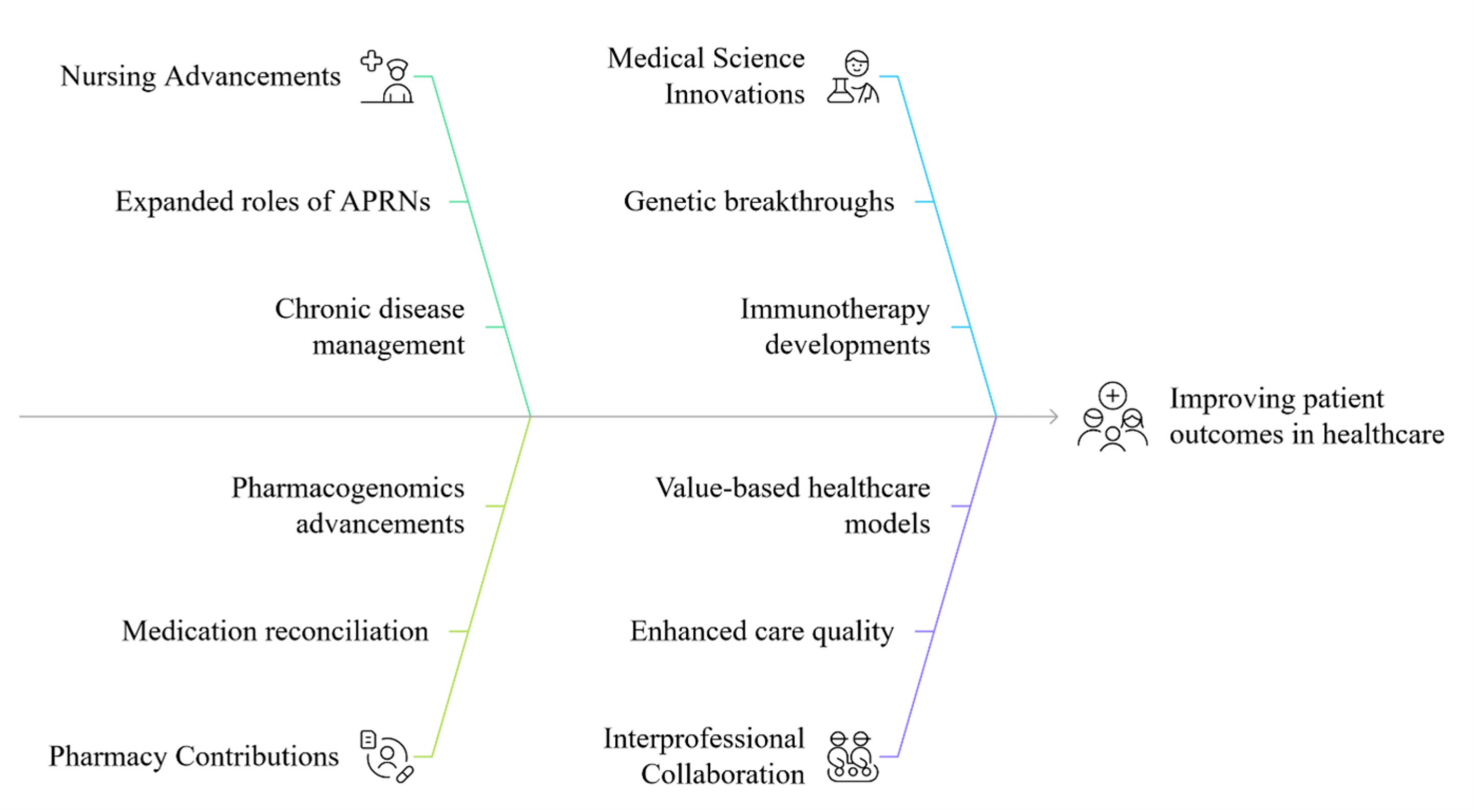
Overview of interdisciplinary advancements. Image credit: All authors.

Medical science remains foundational in driving innovation through breakthroughs in genetics, immunotherapy, and diagnostic technologies. Yet, the effective translation of these advancements into clinical practice depends on their integration with nursing and pharmacy expertise [1]. The convergence of these disciplines is essential for addressing modern healthcare complexities. Interprofessional collaboration (IPC) enhances care quality and safety across settings and is central to the success of value-based healthcare (VBHC) models. As healthcare becomes more integrative, interdisciplinary teamwork will be pivotal in delivering comprehensive, equitable, and high-quality care. Figure 1 illustrates how advancements in nursing, pharmacy, and medical science collectively contribute to improved patient outcomes in healthcare. It highlights the synergistic roles of APRNs, pharmacogenomics, and innovative diagnostics in enabling value-based, coordinated care.

Objectives of the review

The primary objective of this review is to critically synthesize the evolving roles and interprofessional dynamics among nursing, pharmacy, and medical science, with particular emphasis on their collective contributions to advancing clinical practice. By exploring the distinct yet complementary expertise each discipline brings to healthcare, this review seeks to illuminate how their integration fosters improved patient outcomes, enhances the quality and efficiency of care delivery, and facilitates the evolution of collaborative care models. Drawing upon empirical studies and real-world examples, the review also aims to assess the implementation and effectiveness of interdisciplinary approaches across various clinical settings. The research identifies advantages and constraints within existing collaborative structures while providing knowledge to direct both research direction and policy progression regarding worldwide healthcare team practices.

## Review

Building blocks and growth of interprofessional clinical practice

Breaking Down Silos: The Historical Path to Interprofessional Healthcare

The development of healthcare delivery has progressed from self-consistent practitioner operations to integrated medical care delivery models. The three professions of nursing, pharmacy, and medicine started their development independently through distinct training programs and professional boundaries [8]. The initial healthcare systems adopted physician dominance, which restricted nursing activity to bedside tasks, along with medication dispersal duties relegated to pharmacy services. The separate sections of healthcare manifested through specialized knowledge gaps yet produced a fragmented care process and restricted service communications with delivery inefficiencies [9]. 

Several factors throughout time accelerated the development of more collaborative healthcare practices. Advanced medical knowledge and advanced therapeutic approaches made it mandatory to recruit healthcare providers with more extensive patient management expertise. Chronic diseases have become prevalent in society, which demonstrates the inadequacy of treating patients through single professional methods. The expansion of patient-centered approaches, together with rising awareness about team dynamics in safety and results, produced an awareness about interprofessional collaboration's fundamental worth [10,11]. The healthcare sector started to develop policy frameworks and reports that advocated for new integrated care structures that emphasized better teamwork among medical staff members. Interprofessional rounds in hospitals [12] together with collaborative practice agreements in community settings emerged as direct strategies to reduce professional isolation between healthcare providers. The healthcare industry shows signs of progress because medical professionals now understand that excellent patient care depends on uniting multiple professional viewpoints and combining specialized nursing and medical, and pharmaceutical knowledge. Time has shown that disciplinary experience holds vital importance, but professional synergy creates a larger and more meaningful impact in healthcare [13]. The development of interprofessional collaboration in healthcare has progressed through major stages, starting from professional isolation in the pre-1950s until reaching modern digital health and team-based virtual care integration, as shown in Table 1. Multiple policy changes with widening roles and technological advancements over time have developed unified patient-centered healthcare models throughout nursing, pharmacy, and medical science.

**Table 1:** Historical markers in the development of healthcare teamwork between professionals.

Period	Milestone	Impact on practice	References
Pre-1950s	Distinct professional silos in medicine, nursing, and pharmacy	Minimal interdisciplinary communication; hierarchical model with physician dominance	[13]
1950s-1960s	Early recognition of team-based care in psychiatric and geriatric settings	Beginnings of collaborative care in select settings; improved outcomes for vulnerable groups	[10]
1970s-1980s	Introduction of multidisciplinary teams in community care	Growth of coordinated care for chronic illness; rising awareness of team dynamics	[11]
1990s	Expansion of nursing roles and introduction of clinical pharmacy services	Better medication safety, expanded nursing autonomy, and evidence-based practice adoption	[8]
Early 2000s	Formalization of IPE and care coordination models	Joint learning environments emerge; care models align around patient needs and team communication.	[2]
2010s	Policy mandates promoting value-based care and interprofessional collaboration	Expanded APRN and pharmacist roles in chronic disease and medication management	[14]
2020s-Present	Integration of digital health, AI tools, and collaborative virtual care models	Widespread adoption of telehealth, real-time team communication, and increased patient engagement	[15]
Future Outlook	Predicted shift towards fully integrated, tech-enabled, and equity-focused team care	Proactive, data-driven care models with seamless professional integration and patient co-participation	Projected trends

Conceptual frameworks underpinning interprofessional collaboration: Exploring key theories and models that guide effective teamwork

The effective implementation of interprofessional collaboration is not merely a matter of goodwill; it is underpinned by various conceptual frameworks and theoretical models that provide structure and guidance for teamwork. These frameworks provide essential knowledge about successful collaboration through their exploration of fundamental elements and step-by-step processes, and elements that help or impede effectiveness. The Interprofessional Collaborative Competencies Model represents a well-known framework that defines crucial competencies for interprofessional practice through its sections on values and ethics and roles and responsibilities, as well as interprofessional communication and teams and teamwork [8]. The model highlights the need for professionals to respect each other while developing shared role understanding and effective communication methods, and team cohesion to achieve patient-centered objectives [16]. 

According to the Framework for IPE and Collaborative Practice, education and clinical practice remain interconnected, which demonstrates why future healthcare professionals need collaboration preparation to develop effective clinical teamwork [2]. The framework stresses that educational experiences should be shared between healthcare professionals to develop mutual comprehension of their respective roles. Research shows that both organizational culture and teamwork climate directly impact the achievement of interprofessional teams [14]. These conceptual frameworks supply important knowledge that helps researchers comprehend advanced teamwork models in healthcare. Healthcare professionals and researchers can improve their understanding of teamwork dynamics by using these theoretical frameworks to identify successful collaboration factors that lead to better interprofessional practice strategies for patient benefits [10]. 

The innovative edge of nursing practice

Assessing the Influence of Increased APRN Scope on Patient Access and Quality Across Settings

The healthcare environment has brought about substantial growth in the duties performed by APRNs. The healthcare workforce now includes Nurse Practitioners (NPs), Clinical Nurse Specialists (CNSs), Certified Nurse-Midwives (CNMs), and Certified Registered Nurse Anesthetists (CRNAs) who have become essential providers of care and improved patient access in various practice settings. Medical care delivery in primary, acute, and specialty settings has expanded through the integration of APRN practitioners due to patients' complex needs and physicians' shortage, especially in rural and underserved areas [17]. 

Advanced education and clinical training of NPs allow them to deliver complete primary care services that involve diagnosing and treating both acute and chronic illnesses [18]. Advanced nursing providers are essential for healthcare access expansion because they can perform physical evaluations and execute diagnostic testing and prescribe medications to deliver vital healthcare services. CNSs deliver specialized clinical knowledge to particular patient groups and healthcare environments through which they guide evidence-based practice implementation and function as clinical mentors for nursing staff [19]. The combination of their dedication to quality improvement and specialized clinical expertise leads to better patient results in their practice areas. CNMs deliver complete healthcare services to women from prenatal to intrapartum and postpartum stages in healthcare facilities without sufficient physician obstetric services. CRNAs demonstrate their essential role by delivering safe anesthesia care throughout surgical procedures and procedures which enhances service accessibility and efficiency [20]. 

Multiple studies reveal that treating patients with APRNs has resulted in better patient satisfaction, and that sometimes exceeds physician outcomes in handling specific chronic diseases [21]. Studies show that care delivered by nurses has proven successful in treating rheumatoid arthritis and hypertension. These positive outcomes emerge from their patient-focused, holistic healthcare approach combined with their powerful education delivery to patients and their method of working together in collaborative teams. Advanced Practice Registered Nurses will expand their influence in health equity efforts and population-wide access to quality healthcare as healthcare systems evolve [22]. Table 2 demonstrates the complete breakdown of how nursing, together with pharmacy and medical science, work jointly throughout 11 core healthcare practice areas. The table demonstrates the separate but joint functions of each field in creating unified patient-focused healthcare services.

**Table 2:** Expanded roles and collaborative contributions of nursing, pharmacy, and medical science in interprofessional teams.

Domain	Nursing	Pharmacy	Medical science	References
Primary Care	Conducts health assessments, promotes wellness, manages chronic conditions, and educates patients.	Ensures appropriate pharmacotherapy, performs medication reconciliation, and advises on adherence strategies	Diagnoses conditions, initiates treatment plans, and monitors clinical progress.	[23,24]
Acute Care	Monitors vitals, triages, manages emergency protocols, and provides bedside care.	Manages rapid drug dosing, monitors adverse events, and adjusts therapies during acute episodes	Leads emergency interventions, performs surgeries or procedures, and oversees overall case management	[25]
Chronic Disease Management	Coordinates long-term care plans, supports lifestyle changes, and offers psychosocial support	Manages complex medication regimens, reviews polypharmacy risks, evaluates efficacy and safety	Tracks disease progression, modifies treatment pathways, and orders investigations.	[26]
Geriatric Care	Assesses functional status, manages cognitive decline, addresses fall risk, and nutritional needs	Optimizes medication regimens for aging physiology, minimizes drug interactions, and supports deprescribing.	Screens for comorbidities, evaluates frailty syndromes, and oversees multi-specialty referrals.	[27]
Palliative and End-of-Life Care	Provides symptom relief, emotional support, and care planning in alignment with patient values	Manages opioid conversions, anticipates drug interactions, and ensures comfort-focused pharmacology.	Confirms diagnosis, leads discussions on goals of care, supports hospice transitions	[28]
Mental Health Services	Offers therapeutic communication, screens for psychiatric symptoms, and supports treatment adherence.	Adjusts psychotropic regimens, educates on side effects, and reduces polypharmacy risks	Diagnoses psychiatric conditions, prescribes treatment, and evaluates therapy response.	[26]
Maternal and Reproductive Health	Provides prenatal care, childbirth support, and family planning education	Manages medication safety during pregnancy, addresses hormone therapy concerns	Supervises obstetric and gynecological interventions, screens for complications	[19]
Surgical and Anesthesia Support	Prepares patients for procedures, assists in intraoperative care, and monitors post-op recovery	Dose anesthetic agents, manage perioperative pharmacotherapy, and prevent post-surgical infections.	Performs surgeries, plans intraoperative strategy, and leads recovery protocols	[29]
Health Promotion and Education	Delivers public health messaging, facilitates screening and immunization, and educates on risk reduction.	Develops medication education tools, assists in vaccine deployment, and conducts safety surveillance	Designs population-level interventions, advises on preventive screening guidelines.	[30]
Technology and Innovation	Implements telehealth tools, teaches remote monitoring techniques, and supports digital literacy	Analyzes data from smart devices for pharmacologic optimization, integrates decision support tools.	Develops clinical algorithms, contributes to AI diagnostics, and leads translational research initiatives	[31]
Health Equity and Advocacy	Advocates for marginalized populations tailor care to social determinants, address access barriers.	Ensures affordable access to essential medicines, interprets access-related pharmacoeconomic data.	Conducts epidemiological research, designs policies addressing systemic healthcare disparities	[32]
Research and Evidence Translation	Implements best practices from research into clinical workflows, evaluates intervention outcomes.	Conducts drug utilization studies, supports clinical trial implementation, and disseminates pharmacologic findings	Designs trials, synthesizes scientific evidence, and translates findings into practice guidelines.	[33]

Evidence-Based Nursing Interventions and Clinical Innovations: Highlighting Recent Research and Novel Approaches

Practice in nursing evolves from thorough research, together with the creation of new interventions that enhance patient outcomes across different health challenges. Modern nursing practice relies on evidence-based practice (EBP), which guides healthcare professionals to implement tested interventions from systematic research [34]. Modern research in nursing care has produced major advancements, which have resulted in new methods to handle wounds, along with pain and persistent health conditions. Nursing staff experiences in their workplace help identify specific areas where patient care needs innovation and improvement [35]. Nurses act as important connectors between specialist care facilities and primary healthcare settings by setting up shared-care methods for complicated illnesses such as cancer [28]. The knowledge nurses possess about end-of-life care pathways directly affects how well patients and their families experience their care.

The field of wound care benefited substantially from enhanced knowledge of wound healing science and the creation of better dressing solutions and therapy approaches, including negative pressure wound therapy, which enhances healing outcomes and decreases adverse events [4]. The evaluation of new technologies through nursing research and the creation of clinical protocols for their optimal implementation represent essential functions in healthcare practice. Pain management now implements multiple strategies by uniting medications with non-drug treatment methods. Through nursing research, scientists now understand pain processes better and have validated the effectiveness of monitoring-based care and TENS approaches, and individualized pain management plans for enhancing functional outcomes and patient comfort [36]. Evidence-based nursing innovations have improved healthcare management of chronic diseases, which present substantial expenses for health systems. Nurses lead the development of patient education programs, but also the implementation of self-management support interventions, as well as remotely monitored technologies, which build patient empowerment in their healthcare journey. Nurse-led coaching and counseling approaches have proven successful in enhancing medication adherence and lifestyle modifications while controlling disease progression in diabetic and respiratory illness patients among adolescents, according to research [37]. Nurses are utilizing telehealth applications and mobile health technology to provide unlimited healthcare support for patients in their domestic and social settings. Nursing-based innovations that utilize evidence-based approaches from nursing practice are revolutionizing healthcare delivery systems to enhance patient lives among chronic conditions. 

The Role of Nursing in Patient Safety and Quality-Improvement Initiatives: Discussing Nursing-Led Initiatives to Reduce Errors and Enhance Patient Outcomes

The largest group of healthcare professionals who maintain continuous patient contact serve as central elements to ensure both patient safety and healthcare quality enhancement in organizations [38]. The combination of their attentive nature and clinical expertise, and patient advocacy system enables nurses to detect potential risks while stopping adverse events and creating quality improvement strategies for care delivery [39]. All healthcare facilities need nursing-led programs to build safety-oriented cultures and sustain ongoing quality advancement. 

Nurses lead the execution and assessment of protocols that target medication errors because they represent a major preventable harm source. The critical tasks of medication reconciliation and double-checking procedures, as well as patient medication education, are performed by nurses to minimize these risks. The prevention of hospital-acquired infections (HAIs) depends largely on nurses who use evidence-based methods to execute hand hygiene protocols and care procedures for catheters and infection control measures. The continuous observation of patients by nurses who detect early infection indicators leads to prompt interventions that stop serious complications from developing [40]. 

Nurses actively participate in developing initiatives that enhance the quality of patient care in addition to their work in harm prevention. Nurses deliver standardized care pathways while utilizing quality improvement tools like Plan-Do-Study-Act cycles, together with data collection efforts to find improvement opportunities in practice. The evaluation of nursing interventions against patient outcomes relies heavily on nurse-sensitive indicators, which include pressure ulcer rates alongside fall rates and patient satisfaction scores. Nurses actively create an environment of open error reporting, which allows systems and processes to improve continuously for better safety and quality [41]. Nurses must lead and actively contribute to patient safety work as well as quality improvement activities to develop healthcare systems that provide safe and effective patient care. 

Pharmacogenomics evolution in clinical practice introduces individualized medicine because drugs now depend on specific genetic patterns of patients. Researchers from the field study drug response relationships between genetic factors to estimate medication effectiveness, together with safety risks, and how drugs are processed in the body. Pharmacists now play a critical part in the interpretation of pharmacogenomic data because they need to develop practical therapeutic recommendations [42]. The knowledge of patient genetics helps pharmacists decide which drugs to use and sets the correct medication doses while spotting patients most likely to develop harmful drug effects, which leads to better treatment results and safer care for patients.

**Figure 2:**
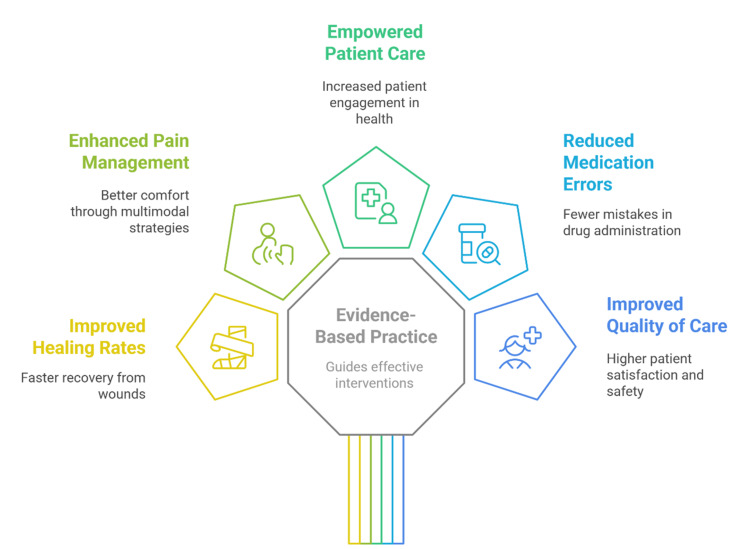
The impact of evidence-based nursing innovations on patient outcomes. Image credit: All authors.

Pharmacists hold a special position to connect genetic testing results with decisions made in clinical practice. Their pharmacology and therapeutic knowledge allow them to explain pharmacogenomic testing implications to patients and other healthcare staff. Pharmacists must explain how genetic testing works while also describing its advantages and restrictions, and then use test results to suggest proper medication changes. Pharmacists take part in healthcare system operations by developing testing protocols for pharmacogenomics, and they ensure correct test usage while integrating results into electronic health records [43]. The future expansion of pharmacist involvement in personalized medicine depends upon decreasing genetic testing costs combined with wider accessibility, because this will lead to better and more exact pharmacotherapy. Figure 2 shows evidence-based nursing innovations enhance patient outcomes, including improved healing results and better pain control, and safer medication use. Evidence-based practice serves as a fundamental guide for interventions that produce improved care quality and empowered patients who achieve better satisfaction.

Advancements in Drug Delivery Systems and Formulation

The pharmaceutical sciences field has developed new advancements which created advanced drug delivery methods that boost both drug efficiency and patient safety levels and enhance therapy compliance. The standard drug delivery methods encounter problems with bioavailability rates and precise drug distribution and need regular dosing that diminishes both treatment effects and medication follow-through [44]. Researchers created various new approaches to overcome these existing challenges.

The features of nanotechnology-based drug delivery platforms let pharmaceutical agents target particular cells or tissues in the body, thus decreasing exposure throughout the body and unwanted side effects. The delivery of drugs directly to tumor sites becomes important in oncology since it creates elevated drug levels at the tumor while protecting untouched parts of the body [45]. Drug development teams create controlled-release and extended-release formulations to establish steady drug concentrations across longer periods while also reducing patient administration requirements and improving their treatment fidelity.

Medical advances include transdermal patches and inhalable formulations, together with implantable drug delivery devices which provide sustained drug delivery for skin-based and respiratory treatments and long-term patient therapy [46]. The field of formulation science now develops better stable medications that preserve their solubility while achieving greater palatability to improve medication usage by patients. Highly developed drug delivery systems, along with formulation innovations, serve to maximize the therapeutic effectiveness of medicines, thus obtaining better patient results together with better life quality. 

Clinical Pharmacy Integration in Multidisciplinary Teams: Impact on Medication Management and Patient Outcomes

Multidisciplinary healthcare teams now integrate clinical pharmacists as essential elements for superior drug management practices, which generate better patient results in diverse treatment settings. Clinical pharmacists now perform active collaboration with physicians and nurses, and other healthcare professionals to optimize pharmacotherapy while resolving drug-related problems [47]. The extensive pharmacological and pharmacokinetic understanding of drug responses allows clinical pharmacists to provide essential support for safe drug use while minimizing costs. Through pharmacoinformatics applications, pharmacists gain superior capabilities to handle difficult drug information and deliver better pharmaceutical care.

Daily rounds in inpatient facilities include clinical pharmacists who contribute drug selection expertise and dosage adjustments and adverse effect monitoring, and drug interaction prevention knowledge [12]. The healthcare team depends on pharmacists to maintain accurate medication lists throughout care transitions, which helps prevent medication errors. Outpatient clinical pharmacists educate patients about medication usage, along with showing them adherence methods and warning them about possible side effects. Pharmacists work together with physicians to optimize medication treatments for hypertension patients and determine appropriate drug choices for complex patients who have dementia [27].

Trusted research exists that shows the positive effects of integrating clinical pharmacists into healthcare delivery. Research evidence demonstrates that multidisciplinary teams, including pharmacists, lead to lower medication-related errors and decrease harmful drug side effects with better patient medicine adherence and cost efficiency, and superior patient satisfaction [5]. Pharmacists make essential contributions to complex patient care because these patients face increased drug-related problems due to multiple health conditions and multiple medications. As healthcare stakeholders embrace team-based care more frequently, clinical pharmacists should expect their integration will grow into the healthcare organization alongside better medication management and superior patient results.

Translating medical innovation into clinical practice

Precision Medicine and Targeted Therapies: Transforming Disease Management

Recent medicine has experienced a revolutionary change through precision medicine because it uses specific patient biological characteristics, which include molecular and genetic, and cellular elements, for customizing diagnostic and therapeutic solutions. The alternative therapy differs from typical uniform treatment methods, which creates opportunities for treatments that can deliver superior outcomes and reduce harmful side effects. Precision medicine relies on complex diagnostic techniques to identify advanced information about personal disease origins. Modern technological advances in genomics and transcriptomics and proteomics, and metabolomics have generated unparalleled knowledge about disease molecular causes, such as cancer, as well as cardiovascular diseases and neurological conditions [48].

The recent advancements in diagnosis established the foundation for developing specific therapeutic approaches that target disease-related molecular targets. The discovery of particular tumor mutations in oncology enabled researchers to create minimal harm targeted drugs like small molecule inhibitors along with monoclonal antibodies that specifically eliminate cancer cells [49]. Other therapeutic areas achieve better patient-centered treatments through possessing knowledge of disease-specific molecular pathways that indicate which groups of patients are most receptive to treatment benefits. Precise clinical implementation of precision medicine depends on strong biomarker development while simultaneously building advanced diagnostic methods and thus integrating complicated data within medical decision systems [33]. Medical scientists and clinicians, and bioinformaticians need to work closely to implement transformative approaches successfully across broad healthcare sectors.

Innovations in Medical Technology and Surgical Techniques

Medical practice has undergone substantial transformation because of quick developments in medical technology and surgical techniques. The recent developments have resulted in medical breakthroughs that enable simpler operations combined with better diagnostic precision and superior treatment achievements with elevated patient rehabilitation speed. MIS stands as a primary advancement in surgical practice because healthcare professionals conduct complex operations through modern instrument access techniques involving laparoscopy and endoscopy, coupled with smaller surgical entry points. The surgical approach of minimally invasive surgery surpasses traditional open surgery because it provides patients with less pain and shorter hospitalization durations as well as reduced infection risks and improved functional recovery times [50]. Through instrument optimization and procedural advancement efforts, MIS now provides safe operation choices to different surgical disciplines.

Surgical robots provide the surgical practice with state-of-the-art tools that ensure precise movements while maintaining manual dexterity. The robotic surgical system grants surgeons better visuals and enhanced movement capabilities, together with better control, which produces exact and minimally invasive procedures for complex surgeries [29]. High-resolution magnetic resonance imaging (MRI) and computed tomography (CT), as well as positron emission tomography (PET) scanner technologies, progress medical imaging diagnostics by supplying enhanced structural and functional details, thus enabling early, precise medical evaluation and better treatment guidance [15]. Medical imaging achieves better analysis results through artificial intelligence (AI) while increasing accuracy in diagnosis and making workflows more efficient. Technological advances continue to extend the limits of what can be performed surgically and diagnosed, which results in better patient outcomes while creating more patient-focused medical services.

Harnessing Cells for Regeneration: Current Status and Potential Breakthroughs

Medical practitioners from different fields unite within regenerative medicine to develop healing therapies through new replacement and regenerative methods that can treat multiple severe ailments. The core element of regenerative medicine requires living cells for treating tissue damage in diseases. Stem cell transplantations provide regenerative medicine with its distinctive cell properties that allow differentiation into numerous cell types, together with engineered cellular platforms that enable therapeutic agent delivery or immunological response management [51].

The clinical use of cell-based treatment approaches has experienced substantial advancement in specified medical situations. The medical community uses hematopoietic stem cell transplantation as an established therapy for treating different blood cancers alongside bone marrow disorders [52]. Medical research explores how mesenchymal stem cells function as potential treatments for inflammatory diseases alongside autoimmune conditions, and they show promise for promoting tissue restorative processes during osteoarthritis and myocardial infarction events [53]. The development of induced pluripotent stem cells (iPSCs), which can be generated from adult somatic cells, offers a potentially unlimited source of patient-specific cells for therapeutic applications, overcoming some of the ethical and immunological challenges associated with embryonic stem cells [54].

While the field of regenerative medicine and cell-based therapies is rapidly evolving, significant challenges remain in terms of scalability, safety, and efficacy for widespread clinical application across diverse conditions. Ongoing research is focused on optimizing cell sourcing, differentiation protocols, delivery methods, and long-term safety monitoring. Nevertheless, the breakthroughs in this field hold tremendous potential to revolutionize the treatment of previously incurable diseases and to restore function in damaged tissues and organs, representing a frontier of medical innovation with profound implications for future clinical practice.

**Figure 3:**
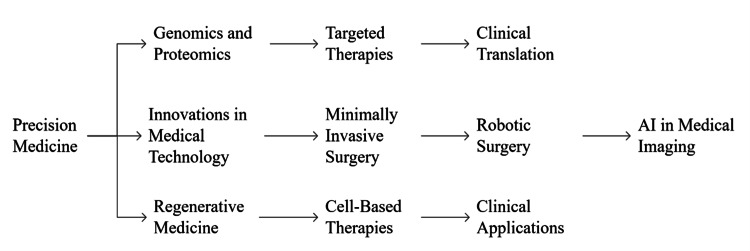
Coordinated interdisciplinary workflow in chronic disease management. Image credit: All authors.

The effective management of chronic diseases, such as diabetes mellitus, cardiovascular disease, and mental health disorders, necessitates a coordinated and integrated approach that transcends the boundaries of individual healthcare disciplines. Collaborative care models, which actively involve nursing, pharmacy, and medicine, have demonstrated significant improvements in patient outcomes, adherence to treatment regimens, and overall quality of life for individuals living with these complex conditions [55]. In diabetes management, for instance, nurses play a pivotal role in patient education regarding self-monitoring of blood glucose, insulin administration techniques, and lifestyle modifications. Pharmacists contribute by optimizing medication regimens, addressing drug interactions, and providing counseling on medication adherence. Physicians are responsible for diagnosis, treatment initiation, and managing complex complications. Integrated care teams, where these professionals communicate regularly and share decision-making, have shown improved glycemic control, reduced hospitalizations, and enhanced patient satisfaction [23]. Figure 3 depicts a streamlined, team-based workflow where nursing, pharmacy, and medical science collaboratively manage chronic disease through interconnected roles.

Similarly, in cardiovascular disease management, nurses provide crucial support in lifestyle counseling, medication adherence, and cardiac rehabilitation programs. Pharmacists ensure optimal antiplatelet therapy, lipid-lowering strategies, and blood pressure control, while physicians manage diagnostic evaluations and interventional procedures. Collaborative models in heart failure clinics, involving nurses titrating medications under protocol, pharmacists monitoring for drug interactions and adverse effects, and physicians overseeing the overall management plan, have been associated with reduced mortality and hospital readmissions [24]. The integration of mental health professionals, including psychiatric nurses and pharmacists specializing in psychotropic medications, alongside physicians, is paramount in addressing the complex needs of individuals with mental health disorders [32]. Collaborative approaches facilitate comprehensive care planning, medication management, and psychosocial support, leading to improved symptom management and functional outcomes [26]. These examples underscore the critical role of shared expertise and coordinated efforts in optimizing care for individuals with chronic conditions. Table 3 displays a comparative overview of the collaborative roles played by various domains of healthcare, highlighting how each discipline contributes uniquely and complementarily within interprofessional teams to enhance patient care and system efficiency.

**Table 3:** Documented outcomes of interprofessional collaboration in clinical settings. CVD, cardiovascular disease

Clinical domain	Nature of collaboration	Documented outcomes	References
Chronic Disease Management	Joint care plans among nurses, pharmacists, and physicians for diabetes, CVD	Improved glycemic control, blood pressure regulation, medication adherence, and reduced hospitalizations	[23,24]
Cardiovascular Care	Collaborative heart failure clinics with nurse-led titration and pharmacist monitoring	Reduced mortality, lower readmission rates, enhanced quality of life	[26]
Oncology and Palliative Care	Nurse navigators, pharmacists, and oncologists co-manage treatment and symptom relief.	Enhanced symptom control, improved patient satisfaction, and better communication of care goals	[36]
Mental Health Services	Psychiatric nurses, clinical pharmacists, and psychiatrists in shared care models	Greater adherence to treatment plans, improved functional outcomes, reduced relapse rates	[26]
Medication Management	Pharmacists are involved in rounds, reconciliation, and therapeutic monitoring.	Decreased medication errors, fewer adverse drug events, cost savings, and higher patient confidence in pharmacotherapy	[47]
Infection Control and AMR Stewardship	Multidisciplinary antimicrobial stewardship programs	Reduction in inappropriate antibiotic use, lower incidence of C. difficile infections, and improved antimicrobial resistance trends	[7,55]
Emergency and Critical Care	Team-based care involving physicians, ICU nurses, and pharmacists	Faster response times, improved triage accuracy, lower ICU mortality, and reduced treatment delays	[25,56]
Health Equity and Community Care	Interdisciplinary outreach teams targeting underserved populations	Increased access to services, culturally tailored interventions, and a reduction in health disparities	[32]
Telehealth and Digital Health	Nurses and pharmacists coordinating remote monitoring and digital consults	Expanded access in rural areas, higher patient engagement, improved continuity of care	[15,30]

Interprofessional Approaches to Acute Care and Critical Illness

In acute care settings, such as emergency departments, intensive care units (ICUs), and surgical suites, the seamless integration of nursing, pharmacy, and medicine is paramount for timely interventions, enhanced patient safety, and optimal outcomes. The rapid and often high-stakes nature of these environments demands effective communication, shared situational awareness, and a unified approach to patient management [11]. In the emergency department, nurses are often the first point of contact, initiating assessments, administering immediate care, and coordinating patient flow. 

Pharmacists play a crucial role in rapid medication dispensing, providing drug information, and identifying potential drug interactions, especially in the context of polypharmacy or unknown medication histories. Physicians are responsible for diagnosis and directing the overall treatment plan. Effective teamwork, characterized by clear roles, open communication, and mutual respect, is essential for efficient resuscitation, accurate diagnosis, and timely initiation of treatment [56].

The ICU environment necessitates highly specialized and coordinated care for critically ill patients. Nurses provide continuous monitoring, administer complex therapies, and are often the first to identify subtle changes in a patient's condition. Pharmacists contribute by optimizing complex medication regimens, ensuring appropriate antimicrobial therapy, and managing parenteral nutrition. Physicians direct the overall medical management, integrating information from all team members to guide treatment decisions. Interprofessional rounds, involving nurses, pharmacists, physicians, and other specialists, facilitate shared understanding of the patient's status and collaborative development of the care plan, leading to improved outcomes and reduced complications [25]. Multiple health professionals, including surgeons and anesthesiologists, along with nurses and pharmacists, need to work together from the preoperative assessment phase to intraoperative care and postoperative recovery in surgical settings to deliver safe patient outcomes. Several healthcare professionals work together through stewardship programs to handle antimicrobial resistance.

Healthcare professionals need to unite their efforts to combat antimicrobial resistance through antimicrobial stewardship programs (ASPs) where nursing and pharmacy practice essential roles [7]. Nurses lead infection prevention and control practices because these practices limit the spread of resistant organisms. Hand hygiene requirements and personal protective equipment protocols, together with preventing physical contact with infectious patients, constitute essential infection prevention methods. Healthcare professionals need to teach patients and their families about infection prevention methods in community environments [57].

Pharmacists have an advanced understanding of antimicrobial pharmacology and pharmacokinetics, as well as resistance mechanisms. The ASP team works to determine the best antibiotics, as well as the correct dosages and administration routes, and treatment lengths. Pharmacists actively participate in antibiotic order reviews by using antibiograms and evidence-based recommendations to make assessments that help identify opportunities for antibiotic use enhancement. Studies show that ASPs, which unite nurses and pharmacists with physicians, achieve substantial success by decreasing antibiotic misuse and Clostridioides difficile infections while preventing antimicrobial resistance development [55]. Educational programs that focus on healthcare providers and patients need to teach them about correct antibiotic usage and proper infection prevention practices as vital elements in the combined fight against AMR.

The Role of IPE and Training in Fostering Effective Collaboration

The education of future healthcare professionals needs basic transformations to develop effective interprofessional collaboration abilities. IPE brings together students from multiple professions through learning about each other and working with one another to achieve better collaboration results and health outcomes [2]. Students from nursing and pharmacy, medicine, and other health professions gain knowledge about their peers' responsibilities through IPE initiatives, which help build essential communication and teamwork competencies for collaborative practice [58].

Implementing successful IPE requires educational approaches that include joint classroom activities with students and simulation-based exercises, and practical clinical teamwork opportunities. The organized learning spaces enable students to develop their communication methods while performing joint decision processes and mastering conflict resolutions within interprofessional settings. IPE leads students to encounter multiple professional cultures while building a common professional language, which represents two essential outcomes [8]. To develop the collaborative behaviors of healthcare professionals who practice while maintaining proficiency, they require continuous multidisciplinary education. Practicing professionals can improve their communication and coordination through initiatives that include team-based learning and multiple-discipline case-based discussions, and simulation-based team training scenarios [30]. Substantial financial investment along with constant interprofessional education will enable the creation of health workers capable of delivering complete, integrated, patient-focused healthcare.

Methodological Considerations and Limitations

This review adopted a narrative synthesis approach to explore advancements in clinical practice through an interdisciplinary lens, emphasizing the roles of nursing, pharmacy, and medical science in driving innovation and improving patient outcomes. The method facilitated the thematic integration of findings from diverse sources-including original research, systematic reviews, meta-analyses, and clinical guidelines-without relying on statistical meta-analysis, allowing for conceptual depth and interpretative analysis. Literature searches were conducted across PubMed, Scopus, and CINAHL using targeted keywords such as “nursing innovation,” “pharmacogenomics,” and “interprofessional teamwork.” Inclusion criteria prioritized peer-reviewed studies with clinical relevance, mostly from the past decade, while foundational older works were considered for context.

Although the methodology provided breadth and flexibility, it carries limitations. Unlike meta-analysis, it does not assess effect sizes or consistency, and findings may be influenced by subjective interpretation and potential selection bias. 

Furthermore, while APRN-led care and clinical pharmacy integration are well-supported, areas like regenerative medicine rely on emerging data [59]. Literature on triadic collaboration among nursing, pharmacy, and medicine remains limited; therefore, the review extrapolated from discipline-specific studies and broader interprofessional research. Acknowledging these limitations underscores the need for further empirical research to evaluate the collective impact of integrated clinical practice across healthcare domains.

Future directions

The future of clinical innovation lies in the integrated efforts of nursing, pharmacy, and medical science to create a cohesive, patient-centered healthcare system. Precision health will require interprofessional collaboration, with pharmacists interpreting pharmacogenomic data to guide personalized therapies and nurses incorporating these insights into holistic care plans that reflect patients’ lifestyles and values. Simultaneously, digital advancements such as artificial intelligence, telehealth, and remote monitoring demand interoperable data platforms and real-time, team-based decision-making. Health disparities require solutions that combine culturally appropriate care practices developed together by interprofessionals who focus on social health factors. Standard interprofessional education (IPE) programs need to be implemented to create future healthcare professionals who respect each other and have a clear role understanding and strong communication skills. Health institutions must work to build collaborative work environments and eliminate professional boundaries between healthcare professionals. The greatest clinical accomplishments emerge through the beneficial union of these health sciences, which create equitable treatment approaches that support individual patient needs.

## Conclusions

This review demonstrates how nursing and pharmacy, together with medical science, actively contribute to developing current clinical practice. Integrated interprofessional collaboration between nursing and pharmacy, and medical science disciplines renders essential service to advance the delivery of healthcare. APRNs expand healthcare accessibility and patient care continuity mainly in underserved areas, while evidence-based nursing practices enhance patient outcomes. Current drug delivery methods are advancing through pharmaceutical sciences, which leads to improved targeted therapy development under clinical pharmacists who maximize both drug safety and effectiveness. Research breakthroughs in precision medicine and minimally invasive technologies, and regenerative therapies have substantially advanced diagnostic capabilities and therapy options.

The development of impactful clinical practice depends heavily on enhancing interdisciplinary teamwork for the future. Sustained integration of healthcare programs presents a necessary solution to control complex medical states while enhancing healthcare organization performance, while fighting antimicrobial resistance as well as health disparities across the globe. The vision requires healthcare systems to back IPE initiatives while applying new technologies and developing organizational settings that emphasize team responsibility and co-decision processes. Medical science and nursing, and pharmacy will unite under a shared objective to create the next transformative healthcare era, which will provide safer patient-centered care that promotes equality for all patients.
